# Long-term trends in mortality by living arrangements and the role of socioeconomic factors, Finland 1991–2020

**DOI:** 10.1093/eurpub/ckaf068

**Published:** 2025-05-09

**Authors:** Ulla K Suulamo, Hanna M Remes, Lasse H Tarkiainen, Pekka T Martikainen

**Affiliations:** Helsinki Institute for Demography and Population Health, Faculty of Social Sciences, University of Helsinki, Helsinki, Finland; Max Planck—University of Helsinki Center for Social Inequalities in Population Health, Helsinki, Finland; International Max Planck Research School for Population, Health and Data Science, Rostock, Germany; Helsinki Institute for Demography and Population Health, Faculty of Social Sciences, University of Helsinki, Helsinki, Finland; Max Planck—University of Helsinki Center for Social Inequalities in Population Health, Helsinki, Finland; Helsinki Institute for Demography and Population Health, Faculty of Social Sciences, University of Helsinki, Helsinki, Finland; Max Planck—University of Helsinki Center for Social Inequalities in Population Health, Helsinki, Finland; Helsinki Institute for Demography and Population Health, Faculty of Social Sciences, University of Helsinki, Helsinki, Finland; Max Planck—University of Helsinki Center for Social Inequalities in Population Health, Helsinki, Finland; Max Planck Institute for Demographic Research, Rostock, Germany

## Abstract

Recent decades have witnessed major changes in living arrangements, potentially impacting their well-established associations with mortality. However, research considering long-term trends in these differentials is scarce. We used individual-level register data on the total Finnish population aged 30 years and over from 1991 to 2020 to examine trends in the association between living arrangements and all-cause, as well as external and alcohol-related mortality. We calculated age-standardized mortality rates, quantified group differentials in absolute and relative terms, and assessed the contribution of socioeconomic factors with Poisson-models. Analyses were conducted separately for men and women in age groups 30–49, 50–69, and 70+. All-cause mortality was consistently lowest among men and women living with a partner. Highest rates were observed in the growing group of individuals living alone or with persons other than a partner or child, who experienced up to a five-fold excess mortality compared to those living with a partner and children. Mortality declined across all living arrangement groups over time. While absolute rate differences mostly narrowed, relative differences widened across all ages. Adjustments for socioeconomic factors somewhat attenuated mortality differentials, with their contribution increasing modestly by the end of the study period. In conclusion, over the past 30 years, relative mortality differences by living arrangement have increased at all ages for both men and women. These widening differentials pose a growing public health burden, particularly for the growing group of individuals living alone. Our results suggest that factors beyond socioeconomic differentiation are contributing to these trends.

## Introduction

Living arrangements are known determinants of mortality in high-income countries, with research consistently indicating those living with a partner as the most advantaged group [1–3]. However, major changes in living arrangements have unfolded over recent decades, resulting in declines in the proportion of two-parent families with children, and in more people living alone, in single-parent households, or in other less traditional settings [4–6]. This growing diversity may be reflected upon mortality disparities across different living arrangements. Yet, there has been little research considering changes in these disparities and the possible sociodemographic drivers behind them.

A long tradition of research has documented lower mortality among married individuals and those living with a partner [7–9], with associations typically more pronounced among men [1, 10, 11], in younger age groups [12], and for deaths related to behaviours, such as accidental, violent, and alcohol-related deaths [13]. It is thought that living with a partner confers health benefits through multiple protective mechanisms, including economic advantages, emotional and social support, and control of harmful behaviours [14, 15]. While these benefits are largely attributed to partnership, other social relationships within households, particularly the presence of children, may also have mortality-protective effects in the working-age population [8]. The association between living arrangements and mortality may, however, also reflect pre-existing differences among individuals selecting into specific living arrangements [16]. For instance, socioeconomic disadvantage may influence union formation [17], dissolution [18], and parenthood [19] with those living alone or with someone other than a partner more often occupying lower socioeconomic positions [20].

Recent changes in living arrangements have raised concerns about their potential health impacts, particularly due to the increasing prevalence of arrangements—such as living alone—that are associated with higher mortality and high-risk health behaviours [8, 21, 22]. While living alone is more common in high-income countries, its prevalence is rising even in regions where it remains low. In Northern America and north-western Europe, one-person households have more than doubled since the 1950s, now making up a third or more of all households [23, 24]. Whether recent changes in living arrangements have implications for how mortality differentials between them are unfolding depends on changes in the health advantages or disadvantages associated with different arrangements or on changes in how people select into them. Evidence suggests that as living arrangements have become more diverse, socioeconomic selection into living with a partner may indeed have intensified [17, 25].

Research on long-term trends in living arrangements and mortality is scarce, but there is some indication of increasing differentials between marital status groups up to the early 2000s [10, 12, 26, 27]. One possible explanation for the increase relates to compositional changes, so that living with a partner is increasingly concentrated among those with higher socioeconomic position or other personal characteristics potentially beneficial to health. While two previous studies [12, 28] have examined whether changes in socioeconomic composition explain the widening mortality gap, they have found limited support for this compositional explanation. However, these studies focused solely on differences between married and non-married individuals.

Using total population register data, we analysed mortality differentials by living arrangements among men and women aged 30 and above from 1991 to 2020. As living arrangements strongly reflect life course stages and change with age, we conducted separate analyses for three age groups: 30–49, 50–69, and 70 and over. Our study addressed two specific research questions: (i) How have mortality differentials by living arrangements changed in the last 30 years for men and women across age groups? (ii) To what extent did these changes arise from changes in the socioeconomic composition of living arrangement groups? In addition to all-cause mortality, we assessed external and alcohol-related deaths, as these behaviour-related causes tend to show particularly pronounced differences by both living arrangement and socioeconomic position and have been key contributors to trends in socioeconomic mortality disparities in Finland in recent decades [8, 13, 21, 29]. We considered both absolute and relative measures of differences, as well as overall mortality levels, to provide a comprehensive view of the trends.

## Methods

### Data

We used Finnish longitudinal population register data compiled at Statistics Finland (permission TK/3343/07.03.00/2023). The study population included individuals aged 30 years and over residing in Finland at the end of baseline years 1990, 1995, 2000, 2005, 2010, and 2015. These data, covering all permanent residents, and containing sociodemographic information from administrative individual-level records were further linked to dates and causes of death in 1991–2020 using personal identification numbers. We followed individuals for 5 years after each baseline year. Altogether, we observed 1 479 576 deaths (9% external and alcohol-related) over 95 842 256 person-years at risk between 1991 and 2020.

We studied all-cause mortality in age groups 30–49, 50–69, and 70 and over, as well as mortality due to external and alcohol-related causes for those under 70. Deaths were classified as external/alcohol-related if the underlying cause was alcohol-attributable disease, injury- or violence-related, or suicide (ICD–10: F10, G312, G4051, G621, G721, I426, K292, K70, K852, K860, O354, P043, Q860, V01–Y89).

We determined individuals’ living arrangements using information on household size and composition. We formed six mutually exclusive groups: (1) living with a partner and child(ren), (2) with a partner, (3) with child(ren), (4) with someone other than partner or child(ren), (5) alone and (6) in an institution or unknown living arrangement. The categories ‘with a partner’ included both married and cohabiting couples. Cohabitation was defined as two non-married persons of different genders living together, who are not siblings, and with an age difference less than 16 years, following the definition of Statistics Finland. Same-sex couples were only classified as living with a partner if their partnership was registered (from 2002) or they were married (from 2017). The category ‘with someone other than partner or child(ren)’ was relatively small across all age groups, including, for example, adults living with their parent(s). Parents living with their child were classified as such regardless of the child’s age or other adults than a partner living in the same household. While we focus on individuals in households, we also present results for those in institutions or with unknown living arrangements.

We explored the extent to which living arrangement differentials in mortality and their change over time was associated with socioeconomic factors. Two indicators were considered: educational attainment and occupation-based socioeconomic position. Information on education was based on the highest achieved qualification and categorized as basic/no qualifications (International Standard Classification of Education ISCED 2011 codes 0–2), secondary (ISCED 3–4), and tertiary (ISCED 5–8). Occupation-based socioeconomic position was grouped into five categories: upper-level employees, lower-level employees, manual workers, self-employed and farmers, and others/unknown. Information on retrospective socioeconomic position was used to categorize the economically inactive by previous occupation, if available. All covariates were measured at the start of each follow-up.

### Statistical analysis

First, we assessed distributional changes in living arrangements by calculating population proportions in each group at the start of the six follow-up periods. We then describe age-, gender-, and living arrangement-specific mortality trends using directly age-standardized mortality rates with 95% confidence intervals (CIs) calculated for six 5-year periods between 1991 and 2020. Separate standard populations for each age group (30–49, 50–69, and 70 and over) were created by aggregating person-years by 1-year age groups across all study periods for men and women combined. From these rates, we calculated absolute differences between individuals living with a partner and children (reference) and other living arrangement groups.

We used Poisson regression to examine relative mortality differences between living arrangements, assess changes over time, and evaluate the effect of socioeconomic factors using data from 1991–95 and 2016–20. Period-specific rate ratios (RR) with 95% CIs were estimated for each living arrangement category using a model that included an interaction term between living arrangement and period. To obtain easily interpretable estimates for living arrangements for both periods, the model was run twice, each time changing the reference period. Specifically, individuals living with a partner and children were used as the reference living arrangement category, first estimating the model with 1991–95 as the reference period and then re-estimating the model again with 2016–20 as the reference. This reparameterization allowed us to estimate period-specific rate ratios while maintaining a consistent model structure for comparability. Model 1 adjusted for age in 5-year categories, while Model 2 further incorporated education and occupation-based socioeconomic position. Analyses were conducted separately for men and women. All-cause mortality was examined separately across all age groups (30–49, 50–69, and 70+), while external and alcohol-related mortality was analysed only for those under 70 due to the small number of such deaths in the oldest age group. All analyses were performed in Stata, version 17.0.

## Results

**Figure 1.**
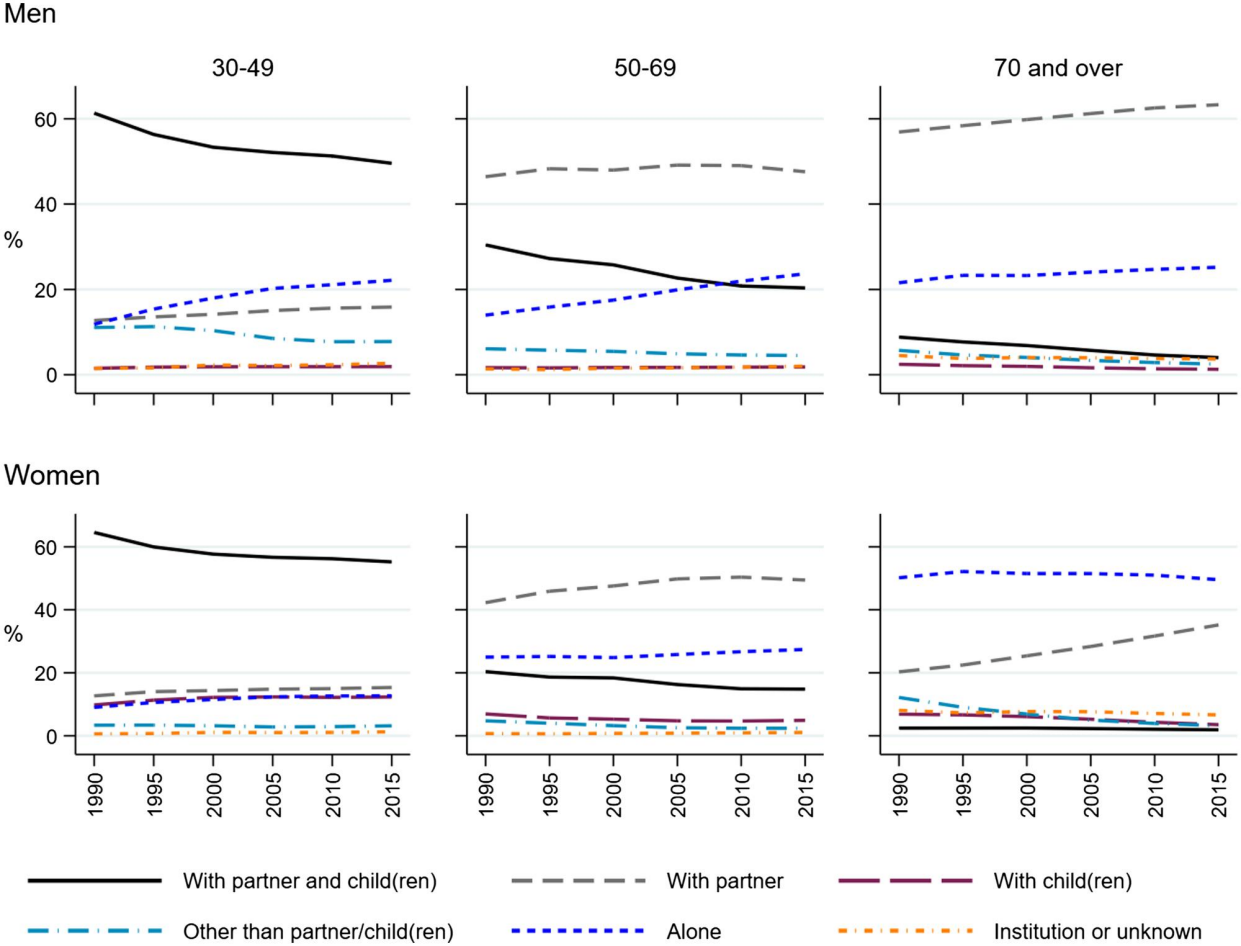
Proportions of population by living arrangement and age group at each baseline year between 1990 and 2015 for men and women aged 30 and over.

Figure 1 shows that while living arrangement distributions varied by sex and age group, the main trends were consistent over time (further descriptive information on the sample in Table S1). A sustained decline was observed in the proportion of individuals living with a partner and children. However, this group remained the largest among the 30- to 49-year-olds, with at least 50% of both men and women living with a partner and children in 2015. Living with a partner increased, particularly among women, and notably at ages 70 and over. Single parenthood increased slightly to 12% among women aged 30–49 by 2015 but stayed below 2% among men. The group of individuals living with someone other than a partner or child was relatively small (<12%) and decreased over time across all age groups. Individuals in institutions or with unknown living arrangements remained stable at under 8%. Living alone increased among men, especially those under 70, while increases were more modest among women.

### Mortality rates by living arrangements

**Figure 2.**
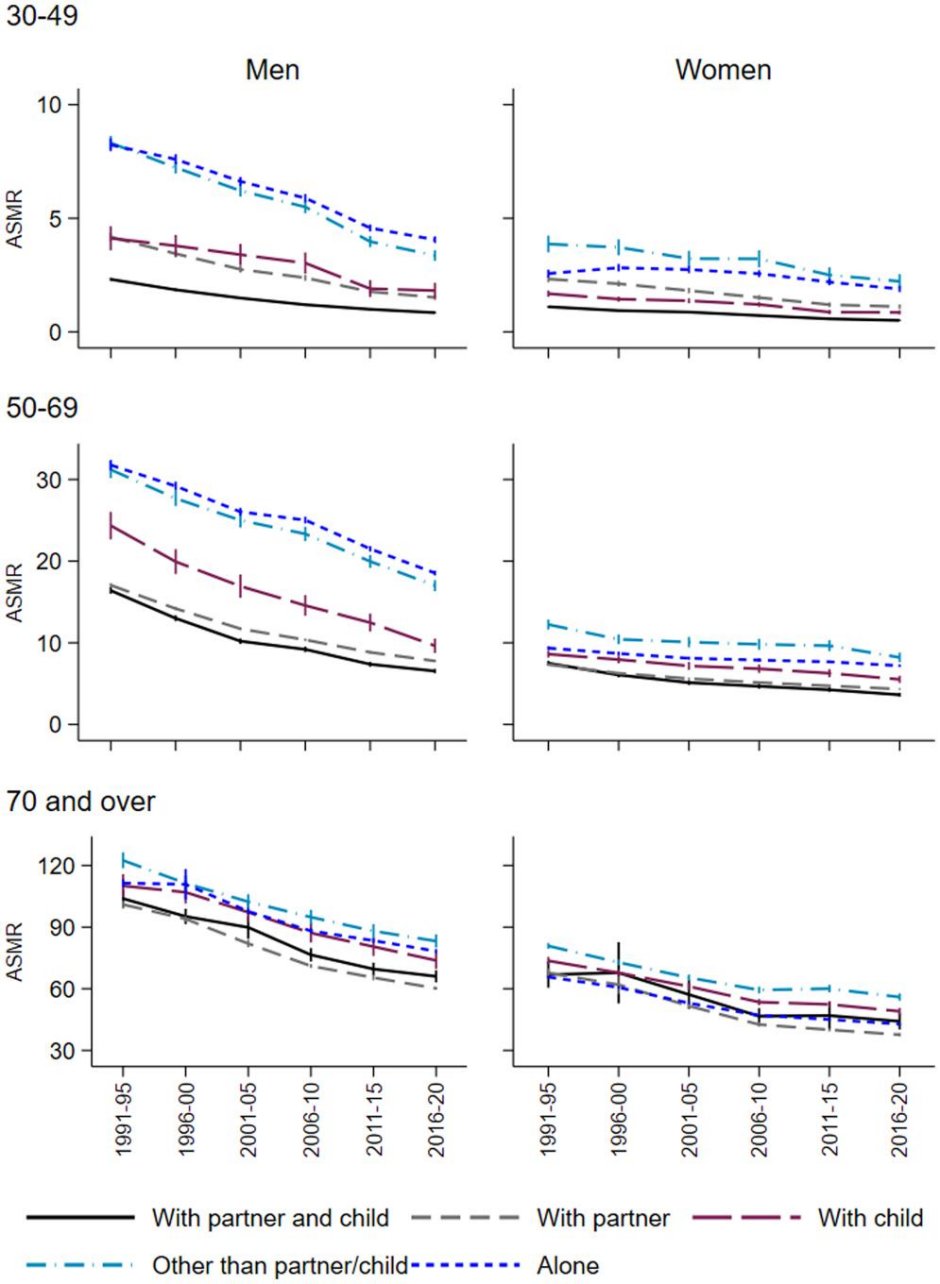
Directly age-standardized mortality rates (per 1000 person-years) by living arrangements, gender, age group, and period; age adjustment using person-years among men and women in the broad age group across all periods as the standard for men and women aged 30 and over.

Over the 30 years, mortality rates were consistently highest among those living alone or those living with others, except for women in the oldest age group, where living alone was associated with lower mortality (Fig. 2; institutions/unknown not included). Below age 70, mortality was lowest among those living with a partner and children, while for those aged 70 and above, rates were lowest among those living with a partner. At younger ages, especially among men, the absolute declines in mortality between 1991–95 and 2016–20 were larger in high-mortality groups, particularly those living alone or with someone other than a partner or children, while among older individuals, largest declines were among those living with a partner. While the absolute gap (the difference in rates) between mortality for those living with a partner and children and those in other living arrangements generally decreased between 1991–95 and 2016–20, it did not do so across all groups. For women aged 50–69 and men aged 70 and over the absolute excess mortality compared to those living with a partner and children even increased, particularly for those living alone (Table S2).

### Relative differences in mortality and the role of socioeconomic factors

**Figure 3.**
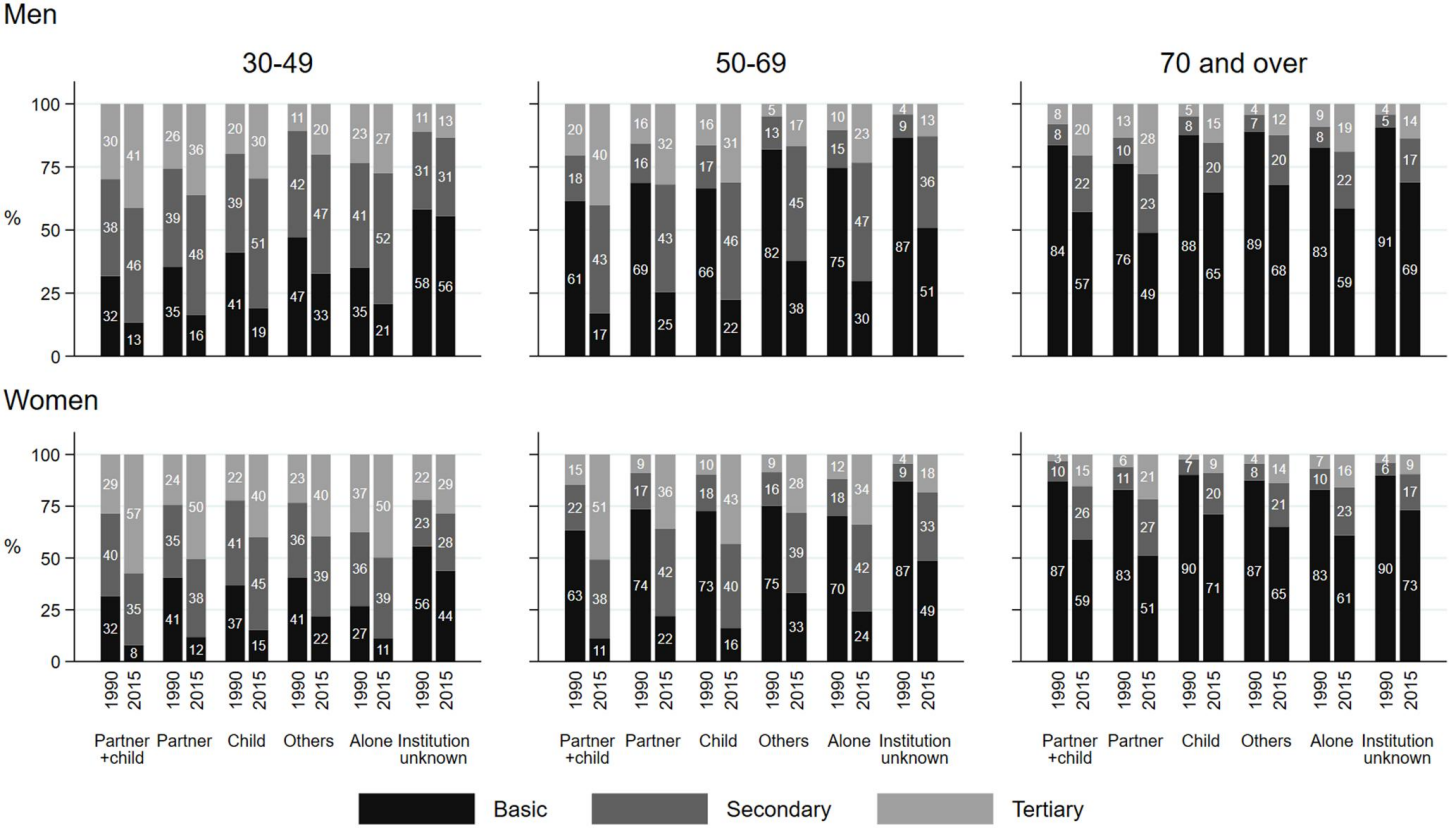
Educational distribution of each living arrangement category by age group in 1990 and 2015 for men and women aged 30 and over.

Those living alone or with others were lower educated and less often held upper-level employee positions than those living with a partner (Fig. 3; see Table S3 for occupation-based social position). Over time, these differences in the socioeconomic composition of the living arrangement groups remained or even widened.

Overall, adjusting for socioeconomic characteristics led to a modest attenuation of the relative excess mortality among those living alone or with others (Table 1). During 1991–95, attenuation was stronger among men. At ages 30–49, excess mortality was reduced by up to 25% after adjustment, depending on the living arrangement category. Similar attenuation was observed among 50- to 69-year-old men but not among women. In the oldest age group, where relative differences by living arrangement were less pronounced, adjusting for socioeconomic factors made little difference.

**Table 1.** Relative all-cause mortality differences by living arrangement in 1991–95 and 2016–20 by age group for men and women aged 30 and over

	**1991–95** ^a^		**2016–20** ^a^	
	Model 1	Model 2	Model 1	Model 2
	RR (CI)	RR (CI)	RR (CI)	RR (CI)
**Men**				
**30–49**				
Partner and children	1.00	1.00	1.00	1.00
Partner	1.78 (1.69, 1.87)	1.67 (1.58, 1.76)	1.75 (1.61, 1.92)	1.62 (1.48, 1.77)
Children	1.78 (1.57, 2.01)	1.64 (1.45, 1.85)	2.12 (1.78, 2.51)	1.93 (1.62, 2.29)
Others	3.60 (3.45, 3.76)	3.02 (2.89, 3.16)	3.99 (3.66, 4.34)	3.15 (2.89, 3.43)
Alone	3.55 (3.41, 3.71)	3.25 (3.11, 3.39)	4.82 (4.53, 5.13)	4.09 (3.84, 4.35)
Institution/unknown	7.34 (6.85, 7.87)	5.76 (5.37, 6.19)	7.26 (6.58, 8.02)	5.13 (4.63, 5.67)
**50–69**				
Partner and children	1.00	1.00	1.00	1.00
Partner	1.13 (1.10, 1.16)	1.11 (1.08, 1.14)	1.32 (1.27, 1.37)	1.27 (1.23, 1.32)
Children	1.60 (1.49, 1.72)	1.56 (1.45, 1.67)	1.54 (1.40, 1.69)	1.47 (1.34, 1.61)
Others	2.08 (2.00, 2.16)	1.91 (1.84, 1.99)	2.87 (2.73, 3.02)	2.52 (2.39, 2.65)
Alone	2.09 (2.03, 2.15)	1.94 (1.88, 2.00)	3.12 (3.00, 3.24)	2.82 (2.71, 2.93)
Institution/unknown	4.29 (4.08, 4.52)	3.66 (3.47, 3.85)	6.67 (6.34, 7.02)	5.58 (5.30, 5.88)
**70 and over**				
Partner and children	1.00	1.00	1.00	1.00
Partner	0.96 (0.93, 0.99)	0.96 (0.93, 1.00)	0.92 (0.89, 0.95)	0.94 (0.90, 0.97)
Children	1.03 (0.98, 1.10)	1.03 (0.97, 1.09)	1.25 (1.18, 1.33)	1.23 (1.16, 1.31)
Others	1.14 (1.09, 1.19)	1.14 (1.09, 1.19)	1.35 (1.28, 1.42)	1.32 (1.26, 1.40)
Alone	1.06 (1.02, 1.09)	1.05 (1.02, 1.09)	1.27 (1.22, 1.32)	1.26 (1.21, 1.31)
Institution/unknown	2.18 (2.09, 2.27)	2.15 (2.06, 2.24)	3.51 (3.36, 3.66)	3.44 (3.30, 3.59)
**Women**				
**30–49**				
Partner and children	1.00	1.00	1.00	1.00
Partner	2.04 (1.90, 2.19)	1.89 (1.76, 2.03)	2.14 (1.92, 2.38)	1.97 (1.77, 2.19)
Children	1.55 (1.42, 1.70)	1.49 (1.36, 1.62)	1.68 (1.49, 1.90)	1.50 (1.32, 1.69)
Others	3.59 (3.24, 3.97)	3.22 (2.92, 3.57)	4.31 (3.70, 5.03)	3.44 (2.95, 4.02)
Alone	2.38 (2.20, 2.57)	2.44 (2.26, 2.64)	3.80 (3.45, 4.18)	3.43 (3.12, 3.77)
Institution/unknown	9.89 (8.62, 11.34)	7.74 (6.73, 8.90)	10.41 (8.93, 12.13)	7.27 (6.22, 8.50)
**50–69**				
Partner and children	1.00	1.00	1.00	1.00
Partner	1.05 (1.01, 1.10)	1.05 (1.01, 1.10)	1.30 (1.23, 1.38)	1.26 (1.19, 1.34)
Children	1.25 (1.17, 1.33)	1.21 (1.14, 1.28)	1.51 (1.37, 1.65)	1.44 (1.31, 1.57)
Others	1.73 (1.63, 1.84)	1.70 (1.60, 1.81)	2.47 (2.26, 2.70)	2.20 (2.01, 2.41)
Alone	1.35 (1.30, 1.41)	1.36 (1.31, 1.42)	2.12 (2.00, 2.25)	2.00 (1.89, 2.13)
Institution/unknown	6.64 (6.16, 7.16)	5.95 (5.51, 6.42)	10.17 (9.43, 10.98)	8.32 (7.70, 8.98)
**70 and over**				
Partner and children	1.00	1.00	1.00	1.00
Partner	1.01 (0.95, 1.06)	1.02 (0.97, 1.08)	0.86 (0.81, 0.91)	0.88 (0.83, 0.93)
Children	1.14 (1.08, 1.21)	1.15 (1.08, 1.21)	1.34 (1.26, 1.42)	1.32 (1.24, 1.40)
Others	1.27 (1.20, 1.34)	1.29 (1.22, 1.36)	1.57 (1.48, 1.66)	1.57 (1.48, 1.66)
Alone	1.03 (0.97, 1.08)	1.05 (1.00, 1.11)	1.15 (1.09, 1.21)	1.16 (1.09, 1.22)
Institution/unknown	2.34 (2.21, 2.47)	2.38 (2.25, 2.51)	3.59 (3.40, 3.80)	3.58 (3.39, 3.79)

Model 1: Adjusted for age; Model 2: Adjusted for age, educational level, and occupation-based socioeconomic position.

a Interaction terms and confidence intervals for the change in rate ratios from 1991–95 and 2016–20 provided in Table S4.

Relative mortality differentials by living arrangement widened over time. Thus, in 2016–20, rate ratios comparing the mortality of those not living with a partner and children to those who were, were higher than in 1991–95, especially at ages 50 and over (Table 1; see Table S4 for interaction terms for the change between periods). This increase in relative mortality disadvantage appeared somewhat more pronounced among women and particularly sharp for those living alone, e.g. an increase from RR 1.35 (CI 1.30, 1.41) to RR 2.12 (CI 2.00, 2.25) for women aged 50–69. In 2016–20, socioeconomic adjustments accounted for up to a third of the relative mortality differences by living arrangement, a slight increase from three decades earlier.

For external and alcohol-related mortality below age 70, age-adjusted relative differences by living arrangement were greater than those observed for all-cause mortality (Table S5). In 1991–95, the relative excess for those not living with a partner and children appeared mostly higher among men. Over time, relative differences increased in both genders, and by 2016–20, the excess in the younger age group appeared even somewhat larger among women. Adjusting for socioeconomic factors reduced the excess external and alcohol-related mortality of those not living with a partner and children similarly to all-cause mortality.

## Discussion

### Main findings

Our findings showed declining mortality across all living arrangement groups over three decades, but persistent excess mortality among those living alone and with others than a partner. While the mortality decline differed in magnitude across sub-groups—being larger in high-mortality groups at younger ages and in low-mortality groups at older ages—the absolute gap between the mortality of individuals living with a partner and children and those in other living arrangements mostly decreased. Relative mortality differences, however, increased. Although socioeconomic adjustment attenuated mortality differentials slightly more in the more recent period, growing socioeconomic differentiation did not account for the growing relative disparities by living arrangement.

### Interpretation of the results

The scarcity of long-term studies on changes in living arrangement differentials in mortality limits direct comparisons with earlier research. Nevertheless, our findings resonate with studies on mortality trends by marital status [1, 10, 12, 26, 30]. These studies, covering high-income countries from around 1950s to 2000, have shown growing relative mortality disparities between married individuals and those in non-married groups. Among them, those also considering overall mortality trends have attributed the developments to slower mortality declines, or even increases, in some of the non-married groups [12, 26]. Our findings for the recent decades showed some differences in the pace of change, but declining mortality across all living arrangement groups. The widening relative differences in mortality observed in this study, are in fact likely to originate in part from the general mortality decline, as relative differences are often larger at lower levels of overall mortality [31].

Lower mortality among those living with a partner has been attributed to a combination of social and health selection, as well as protective effects of health behaviours, economic advantages, and social support [7, 14, 32, 33]. In this study, adjusting for education and occupation-based social class partially attenuated mortality differences between living arrangements, confirming that these differences are partly due to individuals not living with a partner being socioeconomically disadvantaged—consistent with studies based on data from the late 1990s and early 2000s [8, 34]. Despite widening differences in the socioeconomic composition of the living arrangements, including a greater increase in higher education and upper-level occupations among those living with a partner and children, these compositional shifts were only modestly reflected in our results. While socioeconomic factors accounted for a slightly larger share of mortality differences in the more recent period, increased socioeconomic differentiation between living arrangements did not explain the widening relative mortality differences. Similar findings have been reported regarding trends in marital status differences [12, 28], but our study extends this perspective by considering living arrangements that may better reflect the everyday household environment in which people live. Given that socioeconomic factors played only a minor role, other mechanisms likely underlie the increasing disparities.

Previous studies have often reported larger and more consistent mortality differences among men [8, 34–37], commonly concluding they gain greater benefits from cohabiting with significant others. Our results support these findings, most consistently in the 50-69 age group. However, not only the presence of a partner but also that of children may have mortality protective effects among working-age adults [8, 20]. While living with children is believed to encourage healthier behaviours and reduce risky ones, especially for men [38], our findings suggest these benefits may be increasingly important for women. This is further supported by the rising excess mortality from external and alcohol-related causes among women not living with a partner and children, whereas among men, relative differences in these causes showed more modest increases. For men aged 30–49, mortality was similar whether living as a single parent or with a partner, but for women, living with a partner was linked to higher excess mortality than living with a child. This could also reflect differences in selection mechanisms into partnered parenthood, which may affect men and women differently.

Previous research has shown that relative mortality differences between living arrangement categories are greater in younger than older age groups [37]. Our results not only confirm this but also highlight that living arrangements may be becoming increasingly relevant for mortality at older ages. With the growth of the older population, this observation gains particular importance and underscores the need to better understand the impact of living arrangements for health in later life. We found the largest increases in relative mortality differences over time among those aged 70 and over. Notably, compared to men living with a partner, even absolute mortality differences widened for those living alone or with their children.

Living alone has been increasing globally. In Finland—along with other Nordic countries—where welfare state housing subsidies and societal norms such as early independence, informal partnerships, and high divorce rates favour living alone, this trend has been particularly strong, with nearly a half of all Finnish households consisting of just one person in 2023 [39]. Numerous studies have linked living alone to an increased mortality risk and other health adversities [21, 22, 40]. Our analysis showed that in the late 2010s, individuals under age 70 living alone experienced a two- to five-fold excess in mortality compared to those living with a partner and children. Moreover, this group also experienced the largest increases in relative excess mortality over three decades, despite somewhat larger absolute mortality declines among younger men. Although individuals living alone are a diverse group, our analysis indicated that they were, on average, at a disadvantage in terms of education and occupation. While this disadvantage only explained a minor part of their excess mortality, its role became more evident over time, particularly among women. Combined with evidence of a substantial double burden for those living alone with low education or low income [20], this highlights that as single-person households continue to increase, recognizing possible interactions with socioeconomic factors and other vulnerabilities (e.g. loneliness) will become increasingly important.

Findings concerning mortality among older adults living alone are mixed. This has been suggested to indicate that living alone at advanced ages may be selective for those in better health, as serious health issues for those without a partner may lead to an earlier transition to institutional living arrangements. However, selection into living alone depends on both personal and partner health. With increasing survivorship, declining sex differences in mortality, and a reduced risk of widowhood, the dynamics of who ends up living alone—and for how long—have likely changed. While excess mortality among those aged 70+ living alone was low in the 1990s, it has since increased in both men and women. This may reflect shifts in health thresholds for transitioning to institutional care, as suggested by a recent study. Finland’s policy of supporting independent living for older adults for as long as possible—reflecting both cultural values of autonomy and the practical need to manage care for an ageing population—may have contributed to these changes, and thus the older adult population living alone today may include individuals in worse health compared to previous decades. Alternatively, the role of a partner in navigating increasingly fragmented health services may have become more critical over time.

### Methodological considerations

The total population register data used in this study are comprehensive with virtually no loss to follow-up, providing reliable measurements of living arrangements along with demographic and socioeconomic characteristics and mortality over three decades. The reliance on register data eliminates biases typically associated with individuals' retrospective self-reports, making it a valuable resource for examining long-term trends in the association between living arrangements and mortality. While our data offer advantages, certain limitations merit attention.

To provide more streamlined insights on trends, we did not separate cohabiting and married couples. However, we acknowledge that research has shown slightly higher mortality rates among cohabiters compared to married individuals [8, 16]. Our analyses also ignored same-sex couples living in non-registered partnerships. In Finland, children in shared custody can be registered to reside at only one address. Since this is more often the mother, particularly some of the men classified as not living with children may do so part-time.

Not only partnership status and living arrangements but also transitions—particularly those due to life events such as bereavement or couple dissolution—are associated with mortality. As living arrangements diversify, so may their histories. We used a cross-sectional indicator of current living arrangement and did not cover these histories, which should be considered when interpreting our findings. This may be especially relevant for individuals living alone or with others than a partner, whose mortality disadvantage may to a larger extent reflect past experiences rather than the characteristics of their current living arrangement. Living arrangement transitions during the 5-year follow-up may introduce downward bias to our estimates. [Sensitivity analysis with a 2-year follow-up showed qualitatively similar results, though relative mortality differences were somewhat more pronounced in the youngest age group (see Table S6)].

## Conclusion

Living arrangements remain significant in determining mortality outcomes across different stages of life. The advantage of living with a partner has persisted over the past three decades. Encouragingly, mortality has declined across all living arrangements, accompanied by mostly diminishing absolute mortality differentials between groups. However, relative mortality inequalities have widened, particularly affecting those living alone. This growing relative disadvantage is especially concerning as the number of one-person households continues to rise. While the role of socioeconomic factors in explaining mortality differences grew somewhat over time, other factors beyond socioeconomic differentiation appear to be contributing to the growing disparities.

## Supplementary Material

ckaf068_Supplementary_Data

## Data Availability

Data may be obtained from a third party and are not publicly available. Due to data protection regulations of the register holder, the authors are unable to make the data available to third parties. In order to use the data, researchers and research institutions need to apply for a licence that authorizes its use for research purposes. Access to the data can be requested by contacting the register holder. Key points Mortality declined across all living arrangement groups over three decades. Relative inequalities between groups widened over time, especially affecting those living alone, posing a growing public health burden as the number of one-person households rises. Socioeconomic factors explained only a minor part of the widening disparities in mortality between living arrangements.
