# Supplementary material for: Long-term trends in mortality by living arrangements and the role of socioeconomic factors, Finland 1991–2020
Source: Eur J Public Health. 2025 May 9;35(5):814–20. doi: 10.1093/eurpub/ckaf068 (PMC12529274; doi:10.1093/eurpub/ckaf068)
Supplement: ckaf068_Supplementary_Data [file ckaf068_supplementary_data.pdf]

Long-term trends in mortality by living arrangements and the role of socioeconomic factors, Finland 1991–2020

Ulla K Suulamo, Hanna M Remes, Lasse H Tarkiainen, Pekka T Martikainen

References

41. Pasanen TP, Tamminen N, Martelin T, Mankinen K, Solin P. Profiles of subjective health among people living alone: a latent class analysis. *BMC Public Health*. 2021;21:1335.

42. Vigezzi S, Strozza C. Differences in mortality before retirement: The role of living arrangements and marital status in Denmark. *Demographic Res*. 2024;50:515–46.

43. Davis MA, Moritz DJ, Neuhaus JM, Barclay JD, Gee L. Living arrangements, changes in living arrangements, and survival among community dwelling older adults. *Am J Public Health*. 1997;87:371–7.

44. Requena M, Reher D. Residential status and health in middle and late life: a population-based study with new data from Spain. *BMJ Open*. 2020;10:e033330.

45. Korhonen K, Moustgaard H, Murphy M, Martikainen P. Trends in life expectancy in residential long-term care by sociodemographic position in 1999–2018: a multistate life table study of Finnish older adults. *J Gerontol B Psychol Sci Soc Sci*. 2024;79(7):gbae067.

46. Drefahl S. Do the married really live longer? The role of cohabitation and socioeconomic status. *J Marriage Fam*. 2012;74:462–75.

47. Metsä-Simola N, Martikainen P. The short-term and long-term effects of divorce on mortality risk in a large Finnish cohort, 1990–2003. *Popul Stud (Camb)*. 2013;67:97–110.

48. Shor E, Roelfs DJ, Curreli M, Clemow L, Burg MM, Schwartz JE. Widowhood and mortality: a meta-analysis and meta-regression. *Demography*. 2012;49:575–606.

49. Abell J, Steptoe A. Why is living alone in older age related to increased mortality risk? A longitudinal cohort study. *Age ageing*. 2021;50:2019-24.

Table S1. Distribution of study population by living arrangements, age group and gender, in 1990 and 2015; men and women aged 30 and over.

	1990				2015			
	**30–49**	**50–69**	**70+**	**Total**	**30–49**	**50–69**	**70+**	**Total**
**Men**								
Partner and children	76	22	2	100	68	29	2	100
Partner	25	54	21	100	17	54	29	100
Children	51	33	15	100	43	44	13	100
Others	70	23	7	100	57	35	8	100
Alone	49	34	17	100	38	43	19	100
Institution/unknown	47	26	27	100	42	33	25	100
Total	56	33	10	100	40	42	17	100
N	794 273	468 797	147 440	1 410 510	701 779	735 198	303 373	1 740 350
**Women**								
Partner and children	81	18	1	100	75	23	2	100
Partner	25	59	16	100	16	59	24	100
Children	56	28	16	100	61	28	11	100
Others	29	29	42	100	40	34	26	100
Alone	19	38	43	100	17	41	43	100
Institution/unknown	14	11	74	100	18	18	64	100
Total	48	34	19	100	36	41	23	100
N	760 854	536 122	302 888	1 599 864	669 158	760 262	438 390	1 867 810
**Total**								
Partner and children	78	20	2	100	72	26	2	100
Partner	25	56	18	100	17	56	27	100
Children	55	29	16	100	58	31	12	100
Others	53	25	21	100	51	34	15	100
Alone	30	37	34	100	26	42	32	100
Institution/unknown	29	18	54	100	30	26	44	100
Total	52	33	15	100	38	41	21	100
N	1 555 127	1 004 919	450 328	3 010 374	1 370 937	1 495 460	741 763	3 608 160

Note: Values represent row percentages.

Table S2. Deaths, directly age-standardized death rates (per 1000 person years) and absolute difference between the death rate for individuals living with a partner and children and those in other living arrangements in 1991–95 and 2016–2020; men and women aged 30 and over.

	**Deaths**		**Death rate**			**Abs. difference to ref. category**		
	**1991–95**	**2016–20**	**1991–95**	**2016–20**	**Change**	**1991–95**	**2016–20**	**Change**
**Men**								
**30–49**								
Partner and children	5 494	1 485	2.3	0.8	-1,5			
Partner	1 989	753	4.2	1.5	-2,7	1.9	0.7	-1.2
Children	274	142	4.1	1.8	-2,3	1.8	1.0	-0.8
Others	3 120	824	8.3	3.4	-5,0	6.0	2.5	-3.5
Alone	3 520	2 997	8.2	4.1	-4,2	5.9	3.2	-2.7
Institution/unknown	920	535	16.7	6.1	-10,7	14.4	5.2	-9.2
**50–69**								
Partner and children	8 402	3 303	16.4	6.5	-9,9			
Partner	19 672	16 170	17.0	7.8	-9,3	0.6	1.2	0.6
Children	829	509	24.4	9.6	-14,7	8.0	3.1	-4.9
Others	3 761	2 514	31.2	17.0	-14,2	14.8	10.5	-4.3
Alone	9 786	16 068	31.8	18.5	-13,2	15.4	12.0	-3.4
Institution/unknown	1 781	2 676	65.3	39.6	-25,7	48.9	33.1	-15.8
**70 and over**								
Partner and children	4 338	2 940	103.9	66.1	-37,8			
Partner	28 263	43 325	101.0	60.2	-40,8	-2.9	-5.8	-3.0
Children	1 590	1 521	110.1	73.8	-36,3	6.2	7.7	1.5
Others	4 056	2 584	122.5	83.2	-39,3	18.6	17.2	-1.4
Alone	13 665	26 296	111.4	78.3	-33,1	7.5	12.3	4.7
Institution/unknown	5 109	8 491	254.4	234.8	-19,6	150.5	168.7	18.2
**Women**								
**30–49**								
Partner and children	2 492	903	1.1	0.5	-0,6			
Partner	1 131	539	2.3	1.1	-1,2	1.2	0.6	-0.6
Children	617	364	1.7	0.9	-0,8	0.6	0.4	-0.2
Others	447	198	3.9	2.2	-1,6	2.8	1.7	-1.0
Alone	867	795	2.6	1.9	-0,7	1.5	1.4	-0.1
Institution/unknown	224	200	10.5	5.2	-5,3	9.4	4.7	-4.7
**50–69**								
Partner and children	2 946	1 325	7.5	3.6	-3,9			
Partner	8 738	9 335	7.3	4.3	-3,0	-0.2	0.7	0.9
Children	1 621	709	8.6	5.5	-3,1	1.1	1.9	0.8
Others	1 728	750	12.2	8.2	-4,0	4.7	4.6	0.2
Alone	7 508	8 428	9.3	7.2	-2,2	1.9	3.6	1.7
Institution/unknown	904	1 354	45.4	33.5	-11,9	37.9	29.8	-8.1
**70 and over**								
Partner and children	1 411	1 262	66.9	44.2	-22,7			
Partner	12 524	19 986	67.8	37.6	-30,1	0.9	-6.5	-7.4
Children	6 139	4 908	73.6	49.0	-24,6	6.7	4.9	-1.9
Others	14 116	4 962	80.9	56.0	-24,9	14.0	11.8	-2.2
Alone	42 254	55 856	65.7	42.8	-22,9	-1.2	-1.4	-0.2
Institution/unknown	17 436	22 337	187.5	186.3	-1,2	120.6	142.2	21.6

Table S3. Distribution of occupation based socioeconomic position within each living arrangement category by age group in 1990 and 2015; men and women aged 30 and over.

	1990					2015				
	**Upper level**	**Lower level**	**Manual workers**	**Self-employed**	**Other, unknown**	**Upper level**	**Lower level**	**Manual workers**	**Self-employed**	**Other, unknown**
**Men**										
**30–49**										
Partner and children	20	19	42	18	1	28	22	33	14	4
Partner	18	20	48	12	2	24	23	37	10	6
Children	13	17	53	15	1	19	19	41	14	6
Others	6	10	61	19	4	11	15	47	13	14
Alone	16	17	55	10	3	17	19	42	8	13
Institution/unknown	9	9	59	9	14	10	13	42	9	26
Total	18	18	47	16	2	23	21	37	12	8
**50–69**										
Partner and children	15	16	40	29	0	26	19	33	20	2
Partner	13	18	48	21	1	20	19	42	17	2
Children	12	15	49	24	1	18	17	42	20	3
Others	4	7	55	31	3	8	12	54	20	6
Alone	8	12	61	18	2	13	16	53	13	5
Institution/unknown	4	6	62	12	16	8	12	52	15	13
Total	12	15	48	23	1	19	18	43	17	3
**70+**										
Partner and children	6	10	36	48	1	14	14	39	31	2
Partner	11	16	41	31	1	20	20	39	19	2
Children	3	9	37	51	1	10	14	44	30	1
Others	3	7	35	52	3	9	11	43	34	4
Alone	7	13	49	28	2	13	16	49	19	3
Institution/unknown	3	9	51	30	7	10	14	50	21	5
Total	8	14	42	34	2	17	18	42	20	2
**Women**										
**30–49**										
Partner and children	14	46	24	13	2	27	46	14	8	6
Partner	13	46	30	9	2	23	44	18	7	8
Children	12	47	31	7	3	16	45	20	6	12
Others	12	37	30	13	8	18	34	24	8	17
Alone	21	49	23	4	3	22	42	19	5	12
Institution/unknown	12	24	29	9	25	16	24	23	8	29
Total	15	46	25	11	3	24	44	16	7	8

	1990					2015				
	**Upper level**	**Lower level**	**Manual workers**	**Self-employed**	**Other, unknown**	**Upper level**	**Lower level**	**Manual workers**	**Self-employed**	**Other, unknown**
**50–69**										
Partner and children	10	31	34	24	1	24	46	16	11	2
Partner	8	33	41	17	1	17	48	24	10	2
Children	7	28	45	18	1	20	46	22	8	5
Others	6	26	38	27	4	13	36	33	11	8
Alone	9	35	43	12	2	15	45	28	7	4
Institution/unknown	4	15	42	15	23	9	28	35	12	16
Total	9	32	40	18	1	17	46	24	9	3
**70+**										
Partner and children	4	12	42	41	1	9	29	33	27	1
Partner	8	21	41	28	1	14	41	30	14	1
Children	3	11	40	42	4	6	24	44	24	1
Others	4	14	36	40	7	10	28	35	23	5
Alone	6	22	44	23	5	10	37	38	13	2
Institution/unknown	4	15	46	25	10	8	28	43	17	4
Total	6	19	42	28	5	11	37	35	14	2

Note: Values represent row percentages.

Table S4. Interaction terms of changes in rate ratios from 1991–95 to 2016–20 of all-cause mortality and mortality due to external and alcohol related causes by living arrangement and age group; men and women aged 30 and over.

	**All-cause**				**External and alcohol related**			
	**Model 1**		**Model 2**		**Model 1**		**Model 2**	
	**RR**	**95% CI**	**RR**	**95% CI**	**RR**	**95% CI**	**RR**	**95% CI**
**Men**								
**30-49**								
Partner and children	1.00		1.00		1.00		1.00	
Partner	0.99	(0.89, 1.09)	0.97	(0.88, 1.08)	1.02	(0.88, 1.19)	0.97	(0.88, 1.08)
Children	1.19	(0.97, 1.47)	1.17	(0.95, 1.45)	1.02	(0.73, 1.41)	1.17	(0.95, 1.45)
Others	1.11	(1.01, 1.22)	1.04	(0.95, 1.15)	0.97	(0.84, 1.13)	1.04	(0.95, 1.15)
Alone	1.36	(1.26, 1.46)	1.26	(1.17, 1.36)	1.49	(1.34, 1.67)	1.26	(0.17, 1.36)
Institution/unknown	0.99	(0.88, 1.12)	0.89	(0.79, 1.00)	1.03	(0.87, 1.23)	0.89	(0.79, 1.00)
**50-69**								
Partner and children	1.00		1.00		1.00		1.00	
Partner	1.17	(1.11, 1.22)	1.15	(1.10, 1.21)	1.36	(1.20, 1.54)	1.15	(1.10, 1.21)
Children	0.96	(0.85, 1.08)	0.94	(0.84, 1.06)	0.84	(0.63, 1.12)	0.94	(0.84, 1.06)
Others	1.38	(1.29, 1.47)	1.31	(1.23, 1.40)	1.20	(1.03, 1.40)	1.31	(1.23, 1.40)
Alone	1.49	(1.42, 1.56)	1.45	(1.39, 1.52)	1.54	(1.37, 1.74)	1.45	(1.39, 1.52)
Institution/unknown	1.55	(1.45, 1.67)	1.53	(1.42, 1.64)	1.39	(1.16, 1.67)	1.53	(1.42, 1.64)
**70 and over**								
Partner and children	1.00		1.00					
Partner	0.96	(0.92, 1.01)	0.97	(0.92, 1.02)				
Children	1.21	(1.11, 1.31)	1.20	(1.10, 1.30)				
Others	1.18	(1.11, 1.26)	1.17	(1.09, 1.25)				
Alone	1.20	(1.14, 1.26)	1.20	(1.14, 1.26)				
Institution/unknown	1.61	(1.52, 1.71)	1.60	(1.51, 1.70)				
**Women**								
**30-49**								
Partner and children	1.00		1.00		1.00		1.00	
Partner	1.05	(0.93, 1.20)	1.04	(0.92, 1.18)	1.17	(0.92, 1.50)	1.04	(0.92, 1.18)
Children	1.08	(0.93, 1.26)	1.01	(0.87, 1.17)	1.22	(0.92, 1.62)	1.01	(0.87, 1.17)
Others	1.20	(1.00, 1.45)	1.07	(0.89, 1.28)	1.16	(0.78, 1.71)	1.07	(0.89, 1.28)
Alone	1.60	(1.41, 1.80)	1.40	(1.24, 1.59)	1.98	(1.58, 2.49)	1.40	(1.24, 1.59)
Institution/unknown	1.05	(0.86, 1.29)	0.94	(0.76, 1.15)	1.19	(0.76, 1.85)	0.94	(0.76, 1.15)
**50-69**								
Partner and children	1.00		1.00		1.00		1.00	
Partner	1.24	(1.15, 1.33)	1.20	(1.12, 1.29)	1.34	(1.06, 1.69)	1.20	(1.12, 1.29)
Children	1.21	(1.08, 1.35)	1.19	(1.07, 1.33)	1.49	(1.03, 2.14)	1.19	(1.07, 1.33)
Others	1.43	(1.28, 1.59)	1.29	(1.16, 1.44)	1.56	(1.10, 2.21)	1.29	(1.16, 1.44)
Alone	1.57	(1.46, 1.68)	1.47	(1.37, 1.58)	2.03	(1.61, 2.56)	1.47	(1.37, 1.58)
Institution/unknown	1.53	(1.38, 1.70)	1.40	(1.26, 1.56)	1.80	(1.20, 2.70)	1.40	(1.26, 1.56)
**70 and over**								
Partner and children	1.00		1.00					
Partner	0.85	(0.79, 0.92)	0.86	(0.79, 0.93)				
Children	1.17	(1.08, 1.27)	1.15	(1.06, 1.25)				
Others	1.24	(1.14, 1.34)	1.22	(1.13, 1.32)				
Alone	1.12	(1.04, 1.20)	1.10	(1.02, 1.19)				
Institution/unknown	1.54	(1.42, 1.66)	1.51	(1.39, 1.63)				

Model 1. Adjusted for age.
Model 2. Adjusted for age, educational level, and occupation based socioeconomic position.
Note: For example, the estimate of 1.36 for 30–49-year-old men living alone means that their excess mortality increased by 36% between 1991–95 and 2016–20 and can be interpreted as the division of the rate ratio for 2016–20 by the rate ratio for 1991–95 which are presented in Table 1: 4.82 / 3.55 = 1.36.

Table S5. Relative mortality differences by living arrangement in 1991–95 and 2016–20 for mortality due to external and alcohol-related causes; by age group, men and women aged 30 and over.

	**1991–95** [a]		**2016–20** [a]	
	**Model 1**	**Model 2**	**Model 1**	**Model 2**
	**RR CI**	**RR CI**	**RR CI**	**RR CI**
**Men**				
**30–49**				
Partner and children	1.00	1.00	1.00	1.00
Partner	1.99 (1.85, 2.14)	1.87 (1.73, 2.01)	2.03 (1.78, 2.32)	1.88 (1.64, 2.14)
Children	2.11 (1.78, 2.50)	1.92 (1.62, 2.27)	2.14 (1.62, 2.83)	1.92 (1.45, 2.53)
Others	4.18 (3.93, 4.45)	3.42 (3.21, 3.64)	4.07 (3.57, 4.65)	3.15 (2.76, 3.60)
Alone	4.31 (4.07, 4.58)	3.90 (3.68, 4.14)	6.44 (5.86, 7.07)	5.38 (4.89, 5.91)
Institution/unknown	8.35 (7.58, 9.21)	6.49 (5.87, 7.17)	8.62 (7.45, 9.96)	6.03 (5.20, 7.00)
**50–69**				
Partner and children	1.00	1.00	1.00	1.00
Partner	1.24 (1.14, 1.34)	1.19 (1.10, 1.29)	1.68 (1.53, 1.85)	1.61 (1.46, 1.77)
Children	2.29 (1.89, 2.77)	2.21 (1.82, 2.67)	1.92 (1.54, 2.39)	1.82 (1.46, 2.27)
Others	3.77 (3.42, 4.15)	3.45 (3.13, 3.81)	4.52 (4.01, 5.10)	3.97 (3.52, 4.48)
Alone	3.82 (3.52, 4.14)	3.49 (3.22, 3.78)	5.88 (5.37, 6.44)	5.26 (4.80, 5.76)
Institution/unknown	7.20 (6.30, 8.22)	6.09 (5.33, 6.97)	10.04 (8.89, 11.33)	8.46 (7.48, 9.56)
**Women**				
**30–49**				
Partner and children	1.00	1.00	1.00	1.00
Partner	3.12 (2.75, 3.53)	2.87 (2.53, 3.25)	3.65 (2.95, 4.51)	3.29 (2.66, 4.07)
Children	2.28 (1.97, 2.65)	2.14 (1.85, 2.49)	2.80 (2.20, 3.55)	2.37 (1.86, 3.01)
Others	3.90 (3.23, 4.71)	3.39 (2.81, 4.10)	4.51 (3.21, 6.35)	3.39 (2.40, 4.77)
Alone	3.96 (3.48, 4.49)	4.14 (3.64, 4.71)	7.84 (6.50, 9.46)	6.90 (5.73, 8.33)
Institution/unknown	8.84 (6.70, 11.67)	6.43 (4.82, 8.56)	10.50 (7.42, 14.87)	6.57 (4.63, 9.35)
**50–69**				
Partner and children	1.00	1.00	1.00	1.00
Partner	1.41 (1.21, 1.65)	1.38 (1.18, 1.61)	1.88 (1.58, 2.25)	1.79 (1.50, 2.14)
Children	1.17 (0.91, 1.50)	1.12 (0.87, 1.44)	1.74 (1.33, 2.27)	1.65 (1.26, 2.15)
Others	2.36 (1.89, 2.96)	2.28 (1.82, 2.86)	3.69 (2.82, 4.82)	3.14 (2.40, 4.11)
Alone	2.09 (1.78, 2.45)	2.07 (1.76, 2.43)	4.23 (3.55, 5.05)	3.90 (3.27, 4.65)
Institution/unknown	6.61 (4.78, 9.15)	5.74 (4.13, 7.97)	11.93 (9.35, 15.21)	9.32 (7.27, 11.96)

Model 1. Adjusted for age.
Model 2. Adjusted for age, educational level, and occupation based socioeconomic position.
[a] Interaction terms with confidence intervals for the change in rate ratios from 1991–95 to 2016–20 provided in Table S4.
Note: Age group 70+ is not considered due to small number of external and alcohol-related deaths.

Table S6. Relative all-cause mortality differences by living arrangement in 1991–92 and 2016–17; by age group, men and women aged 30 and over.

	**1991–95**		**2016–20**	
	**Model 1**	**Model 2**	**Model 1**	**Model 2**
	**RR CI**	**RR CI**	**RR CI**	**RR CI**
**Men**				
**30–49**				
Partner and children	1.00	1.00	1.00	1.00
Partner	1.97 (1.81, 2.14)	1.85 (1.71, 2.02)	1.96 (1.69, 2.27)	1.81 (1.56, 2.10)
Children	2.15 (1.78, 2.59)	1.99 (1.65, 2.40)	2.53 (1.91, 3.34)	2.30 (1.74, 3.04)
Others	4.00 (3.72, 4.29)	3.34 (3.11, 3.59)	4.33 (3.75, 5.01)	3.41 (2.95, 3.95)
Alone	4.03 (3.76, 4.32)	3.69 (3.45, 3.96)	5.52 (4.96, 6.14)	4.67 (4.20, 5.20)
Institution/unknown	8.46 (7.58, 9.44)	6.61 (5.90, 7.39)	8.67 (7.38, 10.18)	6.05 (5.13, 7.14)
**50–69**				
Partner and children	1.00	1.00	1.00	1.00
Partner	1.09 (1.05, 1.14)	1.07 (1.02, 1.11)	1.35 (1.27, 1.44)	1.31 (1.22, 1.39)
Children	1.63 (1.46, 1.83)	1.58 (1.41, 1.77)	1.62 (1.39, 1.90)	1.54 (1.32, 1.80)
Others	2.04 (1.92, 2.17)	1.88 (1.76, 2.00)	3.11 (2.85, 3.39)	2.72 (2.50, 2.97)
Alone	2.09 (1.99, 2.19)	1.93 (1.84, 2.03)	3.34 (3.14, 3.57)	3.02 (2.83, 3.22)
Institution/unknown	4.64 (4.29, 5.01)	3.94 (3.64, 4.27)	7.91 (7.29, 8.59)	6.61 (6.08, 7.18)
**70 and over**				
Partner and children	1.00	1.00	1.00	1.00
Partner	0.92 (0.88, 0.97)	0.93 (0.89, 0.98)	0.90 (0.84, 0.95)	0.91 (0.86, 0.97)
Children	1.02 (0.94, 1.11)	1.02 (0.93, 1.11)	1.23 (1.12, 1.36)	1.22 (1.10, 1.34)
Others	1.13 (1.05, 1.20)	1.13 (1.05, 1.20)	1.44 (1.33, 1.56)	1.42 (1.31, 1.54)
Alone	1.01 (0.96, 1.07)	1.01 (0.96, 1.06)	1.26 (1.18, 1.33)	1.25 (1.17, 1.33)
Institution/unknown	2.22 (2.09, 2.36)	2.20 (2.07, 2.33)	3.87 (3.63, 4.13)	3.80 (3.56, 4.05)
**Women**				
**30–49**				
Partner and children	1.00	1.00	1.00	1.00
Partner	2.25 (2.00, 2.53)	2.08 (1.85, 2.34)	2.37 (1.98, 2.85)	2.18 (1.77, 2.19)
Children	1.58 (1.36, 1.83)	1.51 (1.31, 1.76)	1.85 (1.51, 2.28)	1.64 (1.32, 1.69)
Others	3.84 (3.26, 4.53)	3.41 (2.89, 4.02)	4.27 (3.25, 5.59)	3.35 (2.95, 4.02)
Alone	2.68 (2.37, 3.04)	2.76 (2.43, 3.13)	4.25 (3.61, 5.00)	3.82 (3.12, 3.77)
Institution/unknown	10.55 (8.45, 13.19)	7.89 (6.28, 9.92)	12.97 (10.15, 16.56)	8.73 (6.22, 8.50)
**50–69**				
Partner and children	1.00	1.00	1.00	1.00
Partner	1.06 (0.99, 1.14)	1.05 (0.98, 1.13)	1.23 (1.12, 1.36)	1.20 (1.19, 1.34)
Children	1.21 (1.10, 1.35)	1.17 (1.06, 1.30)	1.48 (1.27, 1.72)	1.42 (1.31, 1.57)
Others	1.81 (1.64, 2.00)	1.77 (1.60, 1.95)	2.42 (2.08, 2.81)	2.15 (2.01, 2.41)
Alone	1.36 (1.26, 1.46)	1.37 (1.27, 1.47)	2.09 (1.89, 2.30)	1.97 (1.89, 2.13)
Institution/unknown	7.02 (6.23, 7.91)	6.20 (5.48, 7.01)	11.05 (9.78, 12.48)	9.03 (7.70, 8.98)
**70 and over**				
Partner and children	1.00	1.00	1.00	1.00
Partner	1.02 (0.93, 1.11)	1.03 (0.94, 1.13)	0.83 (0.76, 0.92)	0.85 (0.78, 0.94)
Children	1.14 (1.04, 1.26)	1.15 (1.04, 1.26)	1.37 (1.24, 1.52)	1.35 (1.22, 1.50)
Others	1.34 (1.23, 1.47)	1.36 (1.24, 1.49)	1.73 (1.56, 1.91)	1.73 (1.56, 1.91)
Alone	1.01 (0.92, 1.10)	1.03 (0.94, 1.12)	1.12 (1.02, 1.23)	1.13 (1.03, 1.24)
Institution/unknown	2.61 (2.38, 2.85)	2.65 (2.42, 2.90)	4.04 (3.67, 4.43)	4.01 (3.65, 4.41)

Model 1. Adjusted for age.
Model 2. Adjusted for age, educational level, and occupation based socioeconomic position.
